# Job Satisfaction and Psychological Distress among Help-Seeking Men: Does Meaning in Life Play a Role?

**DOI:** 10.3390/bs12030058

**Published:** 2022-02-22

**Authors:** Aiden A. P. Simard, Zac E. Seidler, John L. Oliffe, Simon M. Rice, David Kealy, Andreas Walther, John S. Ogrodniczuk

**Affiliations:** 1 Department of Psychiatry, University of British Columbia, Vancouver, BC V6T 1Z3, Canada; aidensimard104@gmail.com (A.A.P.S.); david.kealy@ubc.ca (D.K.); 2 Orygen, Parkville, VIC 3052, Australia; zac.seidler@movember.com (Z.E.S.); simon.rice@orygen.org.au (S.M.R.); 3 Centre for Youth Mental Health, The University of Melbourne, Parkville, VIC 3010, Australia; 4 School of Nursing, University of British Columbia, Vancouver, BC V6T 1Z3, Canada; john.oliffe@ubc.ca; 5 Department of Nursing, The University of Melbourne, Parkville, VIC 3010, Australia; 6 Department of Clinical Psychology and Psychotherapy, University of Zurich, 8050 Zurich, Switzerland; a.walther@psychologie.uzh.ch

**Keywords:** job satisfaction, meaning in life, psychological distress, men’s mental health, depression, anger

## Abstract

Men’s low job satisfaction has been shown to be associated with greater symptoms of psychological distress. Meaning in life may be an important factor in this relationship, but its role as a mediator has not been reported. The present study investigated meaning in life as a mediator in the relationship between job satisfaction and psychological distress among men. A total of 229 employed Canadian men participated in a cross-sectional survey, completing measures of depression and anxiety symptoms, anger severity, job satisfaction, and the presence of meaning in life. Zero-order correlations were calculated, and regression with mediation analyses were conducted; two models were tested: one for anxiety/depression symptoms and one for anger, as the dependent variables. Both mediation models emerged as significant, revealing a significant mediating effect for job satisfaction on the symptoms of psychological distress (anxiety/depression symptoms, anger) through meaning in life, even while controlling for salient confounding variables including COVID-related impacts. Lower job satisfaction was associated with less meaning in life, which in turn was associated with more symptoms of depression, anxiety, and anger. The findings highlight the importance of job satisfaction in the promotion of a sense of meaning in life among men, leading to improved psychological well-being both inside and outside of the workplace.

## 1. Introduction

There has been growing interest in the promotion of men’s mental health, owing in part to the stubbornly high suicide rates observed in men across most parts of the globe [1]. Efforts to better understand the myriad factors that impact men’s mental health have shone a light on the role of job satisfaction as a key determinant of men’s well-being and mental health [2], but there is a paucity of studies that have further investigated the connection between job satisfaction and mental health among male workers. For the majority of adult men, work is the single activity that occupies most of their waking time. Satisfaction (or lack thereof) with work has been found to have wide-ranging impacts affecting organizations, managers, customers, and workers themselves [3,4,5]. Regarding personal impacts on workers, there is a growing evidence base demonstrating that job satisfaction acts as a significant determinant of men’s mental health and well-being [6,7,8]. For example, low levels of job satisfaction have been shown to be associated with various indices of psychological distress [8,9], including depression [7,10], stress and anxiety [11], and anger [12,13].

While the link between job satisfaction and psychological distress has been well documented [6,9], few studies have investigated the potential mechanisms through which job satisfaction might impact male workers’ mental health. One such factor to consider is meaning in life, which refers to “a sense of coherence or understanding of existence, a sense of purpose in one’s life, the pursuit and attainment of worthwhile goals, and an accompanying sense of fulfillment” [14]. Contemporary models often consider meaning in life within the context of searching for meaning or the presence of meaning [15]. The current article focuses on the presence of meaning in life, referring to the extent to which a person feels their life has meaning, which has been found to have robust associations with mental well-being relative to searching for meaning in life [16]. Several studies have demonstrated that paid work is a primary source of meaning in life [17,18,19,20]. This is especially evident among men [21], as traditional masculine ideals essentialise and overvalue men as the provider or breadwinner, ensuring that the normative foundations of their adult identity and concurrent self-worth are tied up in their employment [22]. Work has also been found to be fundamental to men’s identity [23]. The clearest ramification of this socialized link between men’s work and mental health is the consistent finding that men who are unemployed are significantly more likely to die by suicide than their employed peers [24]. Furthermore, research has highlighted that overall job satisfaction is highly associated with the presence of meaning in life [20]. Additionally, recent literature indicates that meaning in life has a strong association with general well-being [25], depression [26,27], anxiety [28], and anger [29]. Collectively, these findings point to the potential for meaning in life to account for, or mediate, the impact of job satisfaction on men’s psychological distress, though no study published to date has examined this possibility. Furthermore, considering the massive disruptions to work caused by COVID, it seems especially important to examine these constructs within the context of the pandemic.

The present study reports on a preliminary investigation of the association between job satisfaction and psychological distress among Canadian men, and whether meaning in life mediates this association. Psychological distress was conceptualized as symptoms of anxiety/depression and anger, the latter of which studies have shown to feature as a male-type depression symptom [30]. It was hypothesized that lower job satisfaction would be associated with less meaning in life, which in turn would be associated with greater psychological distress. Age, annual income, employment level (full- or part-time), the general impact of COVID on men’s mental health, and COVID-related financial stress were included as covariates in the model being tested in order to account for their potentially confounding effects.

## 2. Materials and Methods

### 2.1. Design and Participants

The data were provided by 229 Canadian men who participated in a cross-sectional survey that was open from 11 May to 30 June 2020, a period of time that coincided with the beginning of the COVID pandemic. The participants were recruited online via the HeadsUpGuys website (https://headsupguys.org; accessed on 21 October 2021), a leading global resource providing tips, tools, information about professional services, and recovery stories to help men fight depression and prevent suicide [31]. The men who expressed an interest in participating were taken to an independent survey site, which was hosted by Qualtrics, where they were presented with the informed consent page. A $500 (CAD) prize draw was offered to incentivize participation. The eligibility criteria included being a minimum age of 18 years old, having online access, being able to read and understand English, self-identifying as male, and residing in Canada. No exclusion criteria were specified. Those providing informed consent to participate then completed the survey online. Only those participants who were employed and provided complete data (N = 229) were included in the present study. The participants’ names were not collected along with the survey data; only IP addresses and study ID numbers were associated with the collected data, which was stored on a secured Canadian server. Ethics approval for the study was granted by the Behavioural Research Ethics Board at the University of British Columbia (H20-01401).

### 2.2. Measures

The measures used in the present study included the Patient Health Questionnaire-4, the PROMIS emotional distress—Anger—short form, the Brief Index of Affective Job Satisfaction, the Meaning in Life Questionnaire, and a demographics form from which the age, annual income, and level of employment data were drawn. Single items were used to assess the general impact of COVID on the respondents’ mental health, and COVID-related financial stress.

The Patient Health Questionnaire-4 (PHQ-4; [32]) is a 4-item self-report questionnaire developed to measure psychological distress associated with symptoms of depression and anxiety [32]. The items are rated on a 4-point Likert scale (0 = “not at all” to 3 = “almost every day”), with the total score representing the severity of the symptoms associated with depression and anxiety. In the current sample, the scale obtained a Cronbach’s alpha of 0.85.

The PROMIS emotional distress—Anger—short form [33] is a 5-item self-report measure that captures the frequency and severity of anger over the past week. The items are rated on a 5-point Likert scale ranging from 1 (Never) to 5 (Always), with the total score representing the severity of anger. In the current sample, the scale obtained a Cronbach’s alpha of 0.89.

The Brief Index of Affective Job Satisfaction [34] is a self-report measure of job satisfaction consisting of four items: “I find real enjoyment in my job”, “I like my job better than the average person”, “Most days, I am enthusiastic about my job” and “I feel fairly well satisfied with my job”. The responses are rated on a five-point Likert scale ranging from 1 = strongly disagree to 5 = strongly agree. Higher total scores reflect greater job satisfaction. In the current sample, the scale obtained a Cronbach’s alpha of 0.93.

The Presence of Meaning subscale of the Meaning in Life Questionnaire (MLQ [29]) was used to assess the degree to which men experienced a sense of meaning in their lives. The respondents answer five items on a 7-point Likert-type scale ranging from 1 (Absolutely True) to 7 (Absolutely Untrue), with higher scores representing a greater presence of meaning in life. In the current sample, the Presence subscale obtained a Cronbach’s alpha of 0.92.

A single item was used to assess the general impact of COVID on one’s mental health, with participants responding to the question “To what extent has COVID-19 affected your mental health?” using a 5-point scale ranging from 1 = very positively to 5 = very negatively.

A single item was also used to assess COVID-related financial stress, with participants responding to the question “To what extent has the COVID-19 pandemic put financial stress on you?” using a 5-point scale ranging from 0 = no stress to 4 = extreme stress.

Information regarding age (treated as a continuous variable), annual personal income ($50,000/year or less vs. more than $50,000/year), and level of employment (full-time vs. part-time) was derived from the demographics form that was used for the survey.

### 2.3. Statistical Analysis

The analyses were performed using SPSS version 25, including the PROCESS macro [35]. Zero-order correlations were computed to examine the simple relationships between all of the variables in the study. Regression with mediation analysis (PROCESS model 4) was employed; in one model using the PHQ-4 total score as the dependent variable, and in the second model using the PROMIS anger total score as the dependent variable. Job satisfaction served as the independent variable, and the MLQ-Presence score served as the mediator. Age, annual income, employment level, the general impact of COVID on one’s mental health, and COVID-related financial stress were included in the model as control variables. Bootstrapped percentile 99% confidence intervals (CI) were estimated using 10,000 re-samples. The statistical significance of an indirect effect of job satisfaction—via the presence of meaning in life—on psychological distress (i.e., symptoms of depression/anxiety, anger) would thus be indicated by the CI not including zero.

## 3. Results

The mean age of the respondents was 38.1 years (SD = 11.67). The majority were Caucasian (76.0%), educated beyond high school (92.6%), employed full-time (84.3%), and had an annual personal income of $50,000 or more (63.8%). Most of the men self-identified as heterosexual (66.8%), were in a relationship (61.6%), and currently lived with their partner/children/extended family (62.4%). The mean PHQ-4 total score was 6.1 (SD = 3.40) and the mean PROMIS anger score was 15.34 (SD = 4.14), which correspond to moderate psychological distress and mild-to-moderate anger, respectively, thus reflecting the help-seeking nature of the sample.

**Figure 1:**
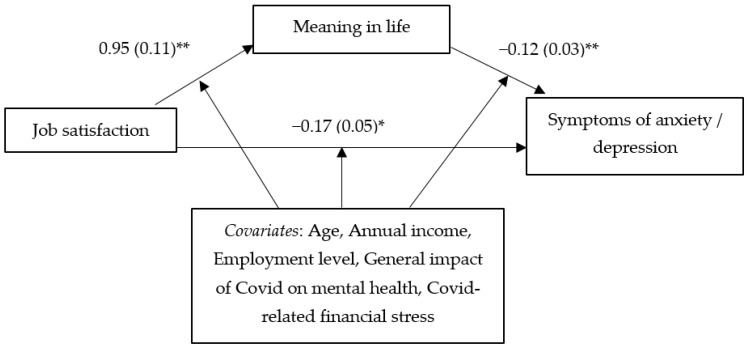
Mediation relationship between job satisfaction, meaning in life, and symptoms of anxiety/depression. * *p* < 0.01; ** *p* < 0.001.

**Figure 2:**
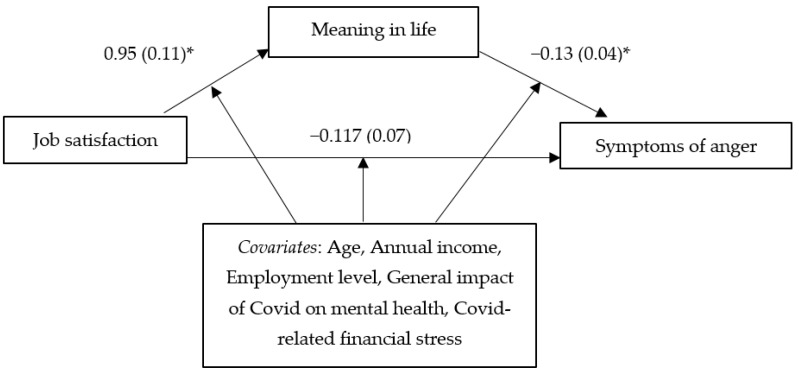
Mediation relationship between job satisfaction, meaning in life, and symptoms of anger. * *p* < 0.001.

Table 1 shows the bivariate correlations among the main study variables. The results of the regression analyses with the PHQ-4 total score serving as the dependent variable (see Table 2, Figure 1), controlling for age, annual income, employment level, the general impact of COVID on one’s mental health, and COVID-related financial stress, indicated that job satisfaction had a significant positive association with meaning in life (*B* = 0.95, *t* = 8.33, *p* < 0.001). In turn, meaning in life had a significant negative association with symptoms of anxiety/depression (*B* = −0.12, *t* = −4.59, *p* < 0.001). Furthermore, the findings showed a significant indirect effect (indicating a mediation effect) of job satisfaction on symptoms of anxiety/depression through meaning in life (*effect* = −0.12, *SE* = 0.03, 99% *CI* = −0.22 to −0.04). This finding revealed that a sense of having less meaning in one’s life—which was more evident for those with lower levels of job satisfaction—was associated with greater symptoms of anxiety/depression. Similar findings emerged from the mediation analysis involving anger as the dependent variable (see Table 2, Figure 2). A significant indirect effect (indicating a mediation effect) was observed for job satisfaction on anger through meaning in life (*effect* = −0.13, *SE* = 0.04, 99% *CI* = −0.25 to −0.02). Lower job satisfaction was associated with less meaning in life (*B* = 0.95, *t* = 8.33, *p* < 0.001), which in turn was related to more anger (*B* = −0.13, *t* = −3.65, *p* < 0.001).

## 4. Discussion

As is consistent with our hypothesis, the present findings demonstrated that men who reported lower job satisfaction were more likely to report reduced meaning in life, which in turn was associated with increased psychological distress (i.e., symptoms of depression, anxiety, and anger). These results emerged even after controlling for age, annual income, employment level, the general impact of COVID on one’s mental health, and COVID-related financial stress. The current findings thus provide preliminary evidence for meaning in life serving as a mediator in the relationship between job dissatisfaction and psychological distress in men.

Our finding of lower job satisfaction being related to less meaning in life is consistent with those of previous studies [19,20,36], with the present study advancing previous work by considering this association in a male-only sample. Given that work is the single activity that occupies most of adult men’s waking time, their dissatisfaction with their work can contribute to them feeling less purposeful with their time and less significant through their actions at work [37]. Indeed, it was implied by the findings of Erdamar and Demirel [38] that when men are dissatisfied with what they do in their job, they are less inclined to be happy and productive in the workplace where they spend most of their day, which contributes negatively to a sense of meaning and satisfaction with life. Additionally, Steger and Dik [15] suggested that when people are more satisfied with their job, it is often accompanied with feeling a “higher purpose” through employment [39], which may contribute to an elevated feeling of meaning in life overall.

The present study also demonstrated that less presence of meaning in life was associated with more symptoms of psychological distress (depression, anxiety, and anger), affirming findings from previous research [25,29,40,41] and extending them to a male-only sample. A lack of meaning in life can contribute to one feeling less significant and agentic, feeling aimless in life [42], and feeling that one’s life goals are unobtainable [43]. Frankl (1963) maintained that the need for meaning is a fundamental human need, and that meaninglessness causes an “existential vacuum” that is characterized by boredom, hopelessness, depression, and/or aggressive behaviour [44,45]. On the other hand, people experiencing meaning in life are better prepared to successfully tackle difficult circumstances, have a strong sense of autonomy and self-determination, and are more satisfied with life [41]. Recent studies have reported that meaning in life moderates risk factors for non-suicidal self-injury [46] and serves as a protective factor against suicidal ideation, suicide attempts, and completed suicides [47]. It is implied by these finding that by supporting men to find meaningfulness in their lives, for example, via meaning-centered counselling [48] or life crafting [49], they may be able to develop a richer life outside of work, thereby mitigating the potentially negative impact of low job satisfaction. This would also help to interrupt a potentially detrimental cycle wherein men’s distress and anger triggered by feelings of worthlessness and an absence of meaning in life lead to increasingly poor performance at work, less job satisfaction and, in turn, more distress.

### Limitations

The findings of the present study should be considered in the context of several limitations. Firstly, the present study was cross-sectional, which limits our ability to identify a causal relationship among the investigated variables. Future research using longitudinal data could increase our understanding of the temporal relationship between job satisfaction, meaning in life, and psychological distress. Secondly, the participants were not asked to elaborate upon specific aspects of their employment or job satisfaction. Future research should include a more comprehensive assessment of these variables in order to provide a more thorough understanding of the factors underlying job (dis)satisfaction and how they relate to meaning in life and psychological distress. Lastly, the present study may lack generalizability due to our sampling method—the participants were recruited through the HeadsUpGuys website, and were therefore all seeking help for or information about depression in men. It is not clear whether the findings of the present study also apply to men who are not seeking help for their mental health.

## 5. Conclusions

Job satisfaction is a key construct in industrial and organizational psychology, and previous research has highlighted its impact on a multitude of organizational outcomes such as job performance, organizational citizenship behaviour, absenteeism, and turnover intentions [50]. The present study extends this work by highlighting the spillover effects of job satisfaction beyond the work environment in contributing to a sense of meaning in one’s life and psychological wellness among men. Organizations should help their male employees to find satisfaction with their work in order to lever happier, more engaged and productive workers through means that extend beyond purely promoting Employee Assistance Programs; instead, they should embed day-to-day practices focused on ascertaining and achieving employee goals, and should provide the positive role-modelling of well-being from executive leadership [51], which also contributes to better economic prosperity for the company [52]. Furthermore, considering that meaning in one’s life is an important contributor to fulfilment, and therefore is both a target and a means to this end, counsellors working with men who are distressed and dissatisfied with their work might be encouraged to help them explore their sense of meaning in life, including the consideration of other sources of meaning outside of one’s job.

## Figures and Tables

**Table 1:** Zero-Order correlations between job satisfaction, meaning in life, anxiety/depression symptoms, and anger.

	1	2	3
1. Job satisfaction			
2. Meaning in life	0.51 *		
3. Anxiety/Depression	−0.43 *	−0.46 *	
4. Anger	−0.29 *	−0.36 *	0.55 *

* *p* < 0.001.

**Table 2:** Results of regression analyses examining the indirect effect of job satisfaction on psychological distress, through meaning in life as a mediator.

**DV: Meaning in Life**	***F***	***R* ^2^**	***b***	**SE**	***t***	***p***
	14.31	0.28				<0.001
Job satisfaction			0.95	0.11	8.33	<0.001
Age			0.01	0.04	0.19	0.85
Annual income			0.52	1.18	0.44	0.66
Employment level			0.87	1.42	0.62	0.54
General impact of COVID on mental health			−0.33	0.64	−0.52	0.60
COVID-related financial stress			−1.00	0.43	−2.31	0.02
**DV: Symptoms of anxiety/depression**	***F***	***R* ^2^**	***b***	**SE**	***t***	***p***
	18.88	0.37				<0.001
Job satisfaction			−0.17	0.05	−3.28	0.001
Meaning in life			−0.12	0.03	−4.59	<0.001
Age			−0.03	0.02	−1.74	0.08
Annual income			−0.27	0.47	0.58	0.56
Employment level			0.02	0.56	0.03	0.98
General impact of COVID on mental health			1.20	0.25	4.77	<0.001
COVID-related financial stress			0.33	0.17	1.88	0.06
**Bootstrap results for indirect effect**			**Effect**	**SE**	**LLCI**	**ULCI**
Job satisfaction via Meaning in life			−0.12	0.03	−0.22	−0.04
**DV: Anger**	***F***	***R* ^2^**	***b***	**SE**	***t***	***p***
	7.12	0.18				<0.001
Job satisfaction			−0.11	0.07	−1.52	0.13
Meaning in life			−0.13	0.04	−3.65	<0.001
Age			0.00	0.02	0.06	0.95
Annual income			−0.33	0.65	−0.51	0.61
Employment level			−0.18	0.78	−0.23	0.82
General impact of COVID on mental health			0.99	0.35	2.82	<0.01
COVID-related financial stress			0.23	0.24	0.98	0.33
**Bootstrap results for indirect effect**			**Effect**	**SE**	**LLCI**	**ULCI**
Job satisfaction via Meaning in life			−0.13	0.04	−0.25	−0.02

Note: The number of bootstrap samples = 10,000; 99% bias-corrected confidence intervals.

## Data Availability

The data used for the present study will be made available by the corresponding author upon request.
